# Supplementary material for: Prevalence and associated factors of delay in seeking malaria treatment among under five children in the Horn of Africa: A systematic review and meta-analysis
Source: PLoS One. 2025 Sep 26;20(9):e0333593. doi: 10.1371/journal.pone.0333593 (PMC12468861; doi:10.1371/journal.pone.0333593)
Supplement: S1 File — (PDF) [file pone.0333593.s001.pdf]

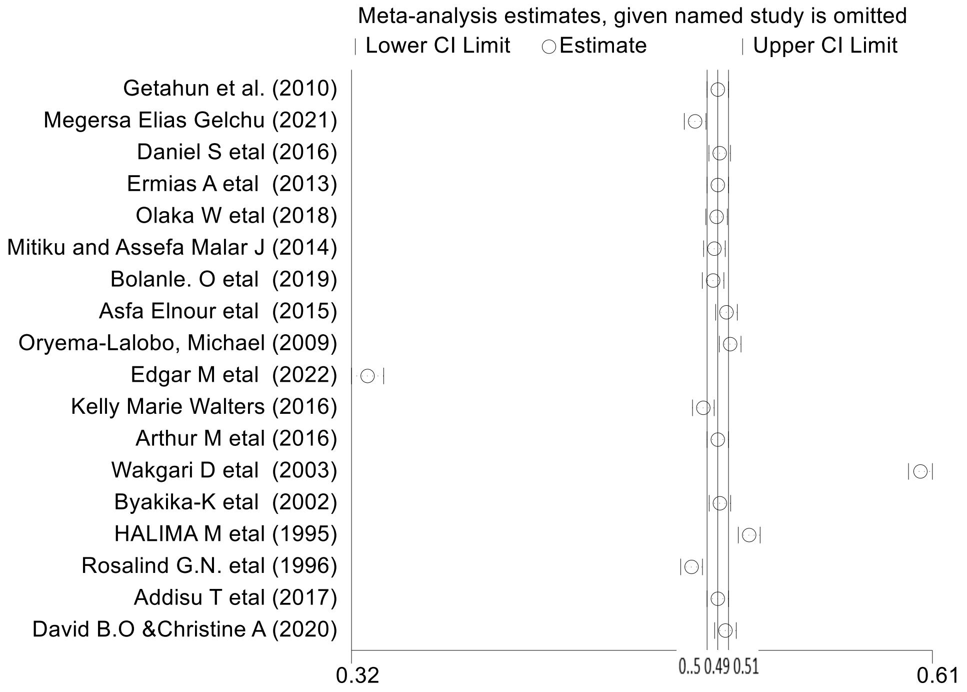


**Supporting file-1: sensitivity analysis for the study of delayed treatment for malaria among children in the horn of Africa.**
