# Supplementary material for: Prevalence and associated factors of delay in seeking malaria treatment among under five children in the Horn of Africa: A systematic review and meta-analysis
Source: PLoS One. 2025 Sep 26;20(9):e0333593. doi: 10.1371/journal.pone.0333593 (PMC12468861; doi:10.1371/journal.pone.0333593)
Supplement: Attachment — Submitted filename: Point-by-point response to reviewers.pdf [file pone.0333593.s007.pdf]

Prevalence and determinants of delay in seeking malaria diagnosis and treatment among under five children in the Horn of Africa: A systematic review and meta-analysis

Muluken Chanie Agimas[1], Mekuriaw Nibret Aweke[2], Berhanu Mengistu[2], Lemlem Daniel Baffa[2], Elsa Awoke Fentie[3], Ever Siyoum Shewarega[3], Aysheshim Kassahun Belew[2], Esmael Ali Muhammad[2]

[1]Department of Epidemiology and Biostatistics, institute of public health, college of medicine and health science, university of Gondar, Gondar, Ethiopia.

[2]Department of human nutrition, institute of public health, college of medicine and health science, university of Gondar, Gondar, Ethiopia.

[3]Department of reproductive health, institute of public health, college of medicine and health science, university of Gondar, Gondar, Ethiopia.

Authors address:

Muluken Chanie Agimas (MCA): mulukensrc12@gmail.com

Mekuriaw Nibret Aweke (MNA): mekunib@gmail.com

Berhanu Mengistu (BM): berhanu5@yahoo.com

Lemlem Daniel Baffa (LDB): lemmidan@gmail.com

Elsa Awoke Fentie (EAF): elsaawoke91@gmail.com

Ever Siyoum Shewarega (ESS): eversiyoum@yahoo.com

Aysheshim Kassahun Belew (AKB): aysheshim121@gmail.com

Esmael Ali Muhammad (EAM): esmaelali34@gmail.com

Corresponding Author

MC: mulukensrc12@gmail.com

To: PLOS ONE

Submission ID: PONE-D-24-08743

Cover Letter for reviewer's comments and authors responses

We would like to thank both the Editorial board members and reviewers for kind comments and suggestions. Accordingly, we have tried to correct and respond to those comments point by point, page by page, and line by line. Therefore, we prepared this author's response, the revised manuscript and the tracked changes of the manuscript. We accept most of your comments and some of your comments are needs justification to be clear for the scientific community.

Sincerely, with best regards!

Muluken Chanie Agimas

ID: https://orcid.org/0000-0003-0808-124X

**Comments from review and editorial board**	**Authors response**
**Editors comment**	**Authors response**
Why is this review limited to studies conducted in the Horn of Africa?	Thank you dear reviewer for you suggestion. Most of the time the horn of Africa is affected by war and instability. Because of this basic health service and infrastructure is very limited. Particularly this sub region is affected by malaria. But there is no

	conclusive evidence about malaria treatment delay and its possible factors. So this study provides an input for decision makers for evidence based decision making.
There is no reference for the first statement in the second paragraph of your introduction, lines 82-84.	Thank you dear editors for you suggestion. We admit the comment and correction has been made accordingly.
In Lines 86-88, the authors wrote: "In African countries, 87 like Kenya, the meta-analysis report showed that only 5% of malaria patients are receiving treatment early 88 (3)". However, the ref (3) provided is not a meta-analysis. Here is your reference 3:<br>Giannone, B., et al., Imported malaria in Switzerland,(1990–2019): A retrospective analysis. 2022. 45: p. 102251.	Sorry dear reviewer. We admit the comment and correction has been made accordingly.
It seems the authors have lost track of some citations. Please, revise all citations and ensure you have referenced them correctly.	Thank you dear reviewer for you suggestion. We can assure that citations are revised accordingly.
Based on the search strategy provided by the authors, I think it's too shallow and perhaps, explains where there were few included studies. No effort at citation tracking was even employed.	Regarding the searching strategy, we all eight authors used the searching engines properly to avoid the possible risk of missing an article. In addition to the searching engines, authors used manual searching of articles in each countries. Additionally, from the retrieved articles, all author review the articles reference to cross check the

	availability of additional eligible articles and tracking of citation.
Please, let your reader know the categories of the JBI scoring you used.<br>Please, credit the CoCoPop and PEO creators whose ideas you applied. Here is one reference;<br>Munn, Z., Stern, C., Aromataris, E., Lockwood, C. & Jordan, Z. What kind of systematic review should I conduct? A proposed typology and guidance for systematic reviewers in the medical and health sciences. BMC Med. Res. Methodol. 18, 5 (2018).	Thank you so much dear reviewer for you interesting suggestion. We admit the comment and correction has been made accordingly.
In lines 134-15, the authors have contradicted themselves by writing: "Studies conducted with Crossectional, case control, cohort and experimental study design were "illegible" for this review. "<br>Please, also give credit to who is due. Here is the reference for the JBI data extraction you used. Please cite it:<br>Munn, Z., Tufanaru, C. & Aromataris, E. JBI's systematic reviews: data extraction and synthesis. Am. J. Nurs. 114, 49–54 (2014).<br>Please, review line 156-160.	Dear reviewer, initially we planned to include all studies done by case control, experimental, cohort, crossectional. But finally we found only articles done by cross-sectional studies.<br>Necessary revision has made for clarity.

State precisely how you calculated each of your study's two outcomes; and what data you used from the primary studies to get that.	Dear reviewer thank you for your feedback. We have clearly wrote this on page 5.
Please cite the reference for your Egger's test and Funnel plot analyses.	Thank you. Now it is revised accordingly.
You have not stated why you used a random effects model meta-analysis only	Thank you so much dear reviewer. Revision has made accordingly.
Your methods section did not note subgroup analysis with reference to the COVID-19 periods.	Dear reviewer it is interesting comment. We tried to mention in the methodology section as sub group analysis by study period which means before COVID pandemic and after COVID pandemic.
There are many references without journal names. Please revise this	Thank you so much dear reviewer. Revision has made accordingly.
In the Discussion, you wrote: "But a study 314 conducted in south-eastern Nigeria was before the COVID-19 pandemic." which study is this and where is its reference?	Dear reviewer the reference is already mentioned in reference number 35.
Please, note that you must provide a reference for all affirmative statements you made.	Thank you dear reviewer. Sentence were fragmented. That is why it seems statement without reference. Now we tried to amend it.
Reviewer two comment	Authors response

This manuscript addresses an important public health issue—delays in seeking malaria treatment for children under five in the Horn of Africa. The study follows a systematic review and meta-analysis approach, synthesizing data to estimate the prevalence of delayed malaria treatment and its associated factors. The topic is relevant, and the authors provide numerical evidence to support their conclusions.	Thank you dear reviewer for your comment.
However, the manuscript requires minor revisions to improve clarity, coherence, and scientific rigor. Key areas needing improvement include language precision, structure, clarity in statistical reporting, and discussion.	Dear reviewer thank you so much for your interesting comments. Your feedback makes our paper more scientifically sound. We feel that the issues you mentioned are needs revisions. So all issues are addressed and amended accordingly.
While the manuscript is understandable, some grammatical errors needs correction and scanty detail should be work on to improve readability<br>The manuscript does not specify the exact search timeframe. The authors should clarify the range of years considered for inclusion.	Dear revewer thank you for your suggestion. We clearly stated the frame of searching in line number 81-82 on page 4. Regarding the range of years considered, we tried to consider all articles conducted in all period of time.
The inclusion/exclusion criteria are not explicitly listed in a structured format.	Thank you so much dear reviewer. We tried to mention the eligibility criteria in structured way on page 5 line 101-110.
While the analysis is rigorous, some results lack proper interpretation.	That is interesting comment. We checked it and amendments were made accordingly.

The study reports odds ratios (ORs) but does not sufficiently explain their meaning in context. Heterogeneity assessment needs more discussion. The I² value should be reported numerically. The discussion should clearly link the results to policy implications and practical recommendations.	Dear reviewer in the interpretation section, we tried to interpret the findings with the numerical value of I² in page 11. But in the discussion part, you are right it needs practical recommendation and policy implications.
Vague recommendations, e.g., "expanding health education" needs improvement. Authors should consider discussions on lack of health system constraints.	Thank you so much dear reviewer. We have made amendments accordingly.
Use consistent tense throughout the manuscript (switching between past and present tenses should be avoided).	Thank you dear reviewer amendment has made accordingly.

Thank you so much for your very impressive scientific contribution.
