# Supplementary material for: Prevalence and associated factors of delay in seeking malaria treatment among under five children in the Horn of Africa: A systematic review and meta-analysis
Source: PLoS One. 2025 Sep 26;20(9):e0333593. doi: 10.1371/journal.pone.0333593 (PMC12468861; doi:10.1371/journal.pone.0333593)
Supplement: Attachment — Submitted filename: Point-by-point_response_to_reviewers_auresp_2.pdf [file pone.0333593.s008.pdf]

Prevalence and associated factors of delay in seeking malaria diagnosis and treatment among under five children in the Horn of Africa: A systematic review and meta-analysis

Muluken Chanie Agimas[1], Mekuriaw Nibret Aweke[2], Berhanu Mengistu[2], Lemlem Daniel Baffa[2], Elsa Awoke Fentie[3], Ever Siyoum Shewarega[3], Aysheshim Kassahun Belew[2], Esmael Ali Muhammad[2]

[1]Department of Epidemiology and Biostatistics, institute of public health, college of medicine and health science, university of Gondar, Gondar, Ethiopia.

[2]Department of human nutrition, institute of public health, college of medicine and health science, university of Gondar, Gondar, Ethiopia.

[3]Department of reproductive health, institute of public health, college of medicine and health science, university of Gondar, Gondar, Ethiopia.

Authors address:

Muluken Chanie Agimas (MCA): mulukensrc12@gmail.com

Mekuriaw Nibret Aweke (MNA): mekunib@gmail.com

Berhanu Mengistu (BM): berhanu5@yahoo.com

Lemlem Daniel Baffa (LDB): lemmidan@gmail.com

Elsa Awoke Fentie (EAF): elsaawoke91@gmail.com

Ever Siyoum Shewarega (ESS): eversiyoum@yahoo.com

Aysheshim Kassahun Belew (AKB): aysheshim121@gmail.com

Esmael Ali Muhammad (EAM): esmaelali34@gmail.com

Corresponding Author

MC: mulukensrc12@gmail.com

To: PLOS ONE

Submission ID: PONE-D-24-08743

Cover Letter for reviewer's comments and authors responses

We would like to thank both the Editorial board members and reviewers for kind comments and suggestions. Accordingly, we have tried to correct and respond to those comments point by point, page by page, and line by line. Therefore, we prepared this author's response, the revised manuscript and the tracked changes of the manuscript. We accept most of your comments and some of your comments are needs justification to be clear for the scientific community.

Sincerely, with best regards!

Muluken Chanie Agimas

Comments from review and editorial board	Authors response
**Reviewer one comment**	**Authors response**
The manuscript conflates "diagnostic delay" and "treatment delay" throughout. These are distinct constructs and must be clearly defined and consistently used.	Thank you for your insightful comment. We acknowledge that "diagnostic delay" and "treatment delay" are distinct constructs, and we appreciate your observation regarding the need for clarity and consistency in their use.<br><br>In response, we have taken the following actions:<br><br>Definitions Added: We have revised the manuscript to clearly define both terms early in the Methods section:<br><br>Diagnostic delay refers to the time between the onset of symptoms and the formal diagnosis.<br><br>Treatment delay refers to the time between diagnosis and the initiation of appropriate treatment which is clearly stated in the method section.<br><br>Consistent Terminology: We carefully reviewed the entire manuscript and revised all instances where these terms were used interchangeably. Each term is now used consistently according to its definition.<br><br>Clarified Discussion: In the Discussion section, we now reflect more precisely on how each type of delay may independently affect patient outcomes.

	We believe these revisions enhance the clarity and rigor of the manuscript and appreciate your guidance in improving its quality.

	conclusive evidence about malaria treatment delay and its possible factors. So this study provides an input for decision makers for evidence-based decision making.
Your search strategy lacks critical detail. Please clearly list the exact search strings, date of last search for each database, and number of hits per database.	Thank you for your valuable feedback. We agree that a transparent and detailed search strategy is essential for the reproducibility and credibility of a systematic review.<br><br>In response, we have revised the Methods section and included the following key details:<br>Exact Search Strings:<br>We now provide the complete search strings used for each database, including Boolean operators, truncations, and subject headings (e.g., MeSH terms), in the Methods on **line 81 to 91**<br><br>Date of Last Search:<br>We have clearly stated the exact date on which each database was last searched (line 81-82).

	We now list the number of records retrieved from each database prior to deduplication.<br>An example excerpts now included in the manuscript is as follows:<br>A complete table summarizing search details for all databases (e.g., Google, Google Scholar, PubMed/Medline, EMBASE, SCOPUS databases, and the published article's reference) has also been added to ensure transparency and replicability.
The use of only English-language articles should be acknowledged as a limitation due to potential language bias.	Sorry dear reviewer. We admit the comment and correction has been made accordingly.
It is unclear why a 50% threshold on the JBI tool was used to assess study quality. Please provide a rationale or reference.	Thank you for highlighting this important point. We acknowledge the need to provide a clear rationale for the use of a 50% threshold in the application of the JBI critical appraisal tool for assessing study quality. In response, we have revised the Methods section to include the following explanation:<br><br>We applied a 50% threshold as a pragmatic cut-off to distinguish between higher- and lower-quality studies, consistent with approaches used in prior systematic reviews utilizing the JBI tool (e.g., [Author, Year]; [Author, Year]). Studies meeting ≥50% of the applicable JBI criteria were considered to be of "moderate to high" quality, while those scoring below were considered "low" quality. This threshold provides a balance between methodological rigor and inclusivity, particularly in fields where higher-quality studies are limited.

	Regarding the searching strategy, we all eight authors used the searching engines properly to avoid the possible risk of missing an article. In addition to the searching engines, authors used manual searching of articles in each countries. Additionally, from the retrieved articles, all author review the articles reference to cross check the

The reported I^2 of 98.8% indicates substantial heterogeneity, yet the implications are not critically discussed. Please address the impact of this heterogeneity on the validity of your pooled estimate.	Thank you for this important observation. We agree that an I^2 value of 98.8% indicates substantial heterogeneity, and we appreciate the opportunity to clarify its implications for our findings. In response, we have revised the Results and Discussion sections to explicitly address the impact of this heterogeneity:<br>The observed I^2 value of 98.8% suggests considerable variability among the included studies that is not due to chance. This level of heterogeneity may stem from differences in study populations, intervention types, outcome measures, or study quality. As such, the pooled estimate should be interpreted with caution.<br><br>To account for this, we used a random-effects model, which assumes that the true effect size may vary between studies. However, even with this model, the high I^2 indicates that the pooled estimate may not reflect a single underlying effect and should be considered a summary of diverse effects rather than a precise estimate.

	We have also conducted subgroup analyses and sensitivity analyses to explore potential sources of heterogeneity though substantial heterogeneity persisted, suggesting that it may be inherent to the variability in study contexts and designs. We believe these additions improve the transparency and critical interpretation of our meta-analytic findings.
While subgroup analyses are conducted, no meta-regression was attempted to explore multiple sources of variability. Please consider this or explain its omission	Thank you for this valuable comment. We agree that meta-regression can be a powerful tool for exploring potential sources of heterogeneity across studies.

While subgroup analyses are conducted, no meta-regression was attempted to explore multiple sources of variability. Please consider this or explain its omission	Thank you for this valuable comment. We agree that meta-regression can be a powerful tool for exploring potential sources of heterogeneity across studies.<br><br>We considered conducting a meta-regression; however, we did not proceed for the following reasons, which are now clarified in the revised Methods and Discussion sections:<br><br>Although meta-regression was considered to explore sources of heterogeneity, we did not perform it due to the limited number of included studies (n = X). As recommended in the Cochrane Handbook for Systematic Reviews of Interventions, **a minimum of 10 studies per covariate is typically required to ensure reliable and valid results in meta-regression.** Conducting meta-regression with fewer studies may lead to unstable estimates and increased risk of type I or II errors.<br>Instead, we performed subgroup analyses based on key study-level variables to explore potential moderators of effect size.<br>We acknowledge the limitations of not performing a meta-regression and have included this as a point for future research, particularly if more studies become available.

• Definitions for categories such as "expensive" and "optimum" transport cost, or "middle income," are unclear. Clarify how these were determined and whether definitions were consistent across included studies.	Thank you for this helpful observation. We agree that clear definitions for terms such as "expensive," "optimum" transport cost, and "middle income" are essential for transparency and interpretability.

	In response, we have revised the Methods and Results sections to include the following clarifications:<br><br>Definition of "Expensive" and "Optimum" Transport Costs:<br><br>These categories were defined based on thresholds reported or implied within the included studies themselves. Where studies specified transport costs as a barrier or facilitator, we categorized:<br><br>"Expensive" as any transport cost described as unaffordable, high relative to daily income, or explicitly reported as a reason for missed visitation.<br><br>"Optimum" as transport costs described as reasonable, subsidized, or not cited as a barrier to visitation.<br><br>In cases where subjective language was used (e.g., "high cost," "reasonable fare"), we applied a consistent interpretation across studies based on local income context.<br><br>Definition of "Middle Income":<br><br>We adopted the World Bank's classification at the time of the study (based on Gross National Income per capita) to define country income levels (e.g., low, lower-middle, upper-middle, high income). A reference and explanation have been added to the Methods section.<br><br>Consistency Across Studies:<br><br>As the included studies varied in how they reported these variables, we have added a note in the Limitations section acknowledging that while we aimed for

	consistent categorization, some variability in reporting may affect how categories were applied across contexts.<br><br>These clarifications have been integrated into the manuscript to improve clarity and reproducibility.
Figures are not integrated within the main manuscript. Please ensure that all referenced figures (e.g., forest plots, funnel plots) are embedded and clearly labeled.<br>• Table 3 is difficult to read due to formatting issues. Please revise it to ensure clarity and alignment of variables, estimates, and confidence intervals.	Thank you for your helpful feedback regarding the presentation of figures and tables.<br><br>Integration of Figures:<br><br>We have now embedded all referenced figures, including forest plots and funnel plots, directly within the main manuscript at the appropriate locations (as per journal guidelines). Each figure is now:<br><br>Clearly labeled (e.g., Figure 1: Forest Plot of Overall Effect Size),<br><br>Sequentially numbered,<br><br>Properly cited within the text (e.g., "...as shown in Figure 2").<br><br>Revision of Table 3:<br><br>We have completely revised Table 3 to improve its readability and alignment. Specific improvements include:<br><br>Clear separation of columns for variables, effect estimates, confidence intervals, and p-values.<br><br>Consistent formatting of numerical data (e.g., decimal places, CI brackets).<br><br>Adjusted column widths and alignment to prevent text wrapping or overlap.

	A descriptive title and footnotes added for clarity.<br><br>We believe these revisions enhance the readability and professional presentation of the manuscript. Thank you for helping us improve its clarity.
. Language and Typographical Issues<br>• The manuscript contains numerous grammatical, typographic, and formatting issues that impede readability. Common issues include incorrect punctuation, inconsistent capitalization, subject–verb agreement errors, and awkward phrasing.<br>• We recommend a thorough language and editorial review, preferably with assistance from a professional editor or fluent English speaker.	Thank you for your constructive feedback regarding the language and formatting of the manuscript.<br><br>We acknowledge that the manuscript contained grammatical errors, typographical issues, and instances of unclear phrasing that may have affected readability. In response:<br><br>The entire manuscript has undergone a thorough language and editorial review, including correction of:<br><br>✓ Subject–verb agreement errors<br>✓ Incorrect punctuation and inconsistent capitalization<br>✓ Typographical mistakes<br>✓ Awkward or unclear phrasing<br><br>We also sought assistance from a professional editor / native English speaker to ensure clarity, coherence, and academic tone throughout the text.<br><br>We believe these revisions have significantly improved the readability and professionalism of the manuscript, and we thank you again for highlighting this important issue.
Contextualization and Literature Integration<br>• The discussion lacks sufficient comparison with existing literature beyond a few regional studies. Please strengthen this section by	Thank you for this valuable suggestion. We agree that broader integration of relevant global and regional literature is essential to

incorporating relevant global and regional findings to contextualize your results.<br>• Assertions regarding the impact of COVID-19 on treatment delays should be supported by references or data.	contextualize our findings and enhance the depth of the Discussion section.<br><br>In response, we have undertaken the following revisions:<br><br>Expanded Comparison with Existing Literature:<br><br>We have strengthened the Discussion by incorporating findings from a wider range of international and regional studies, including those from: Sub-Saharan Africa and High-income countries, where applicable.<br><br>These studies provide important comparative perspectives on the determinants of diagnostic and treatment delays, the role of healthcare access, and the influence of socio-economic factors. We have specifically highlighted areas where our findings align with, or diverge from, previous research and offered possible explanations.<br><br>COVID-19–Related Assertions Supported by Evidence:<br><br>We have revised all statements related to the impact of COVID-19 on treatment delays to ensure they are evidence-based. References have been added.<br><br>Example added reference:<br><br>World Health Organization (2021). The impact of the COVID-19 pandemic on noncommunicable and communicable disease resources and services: results of a rapid assessment. WHO.<br><br>We believe these additions have substantially improved the manuscript's contextual

	richness and global relevance, and we thank the reviewer for prompting this enhancement.
Standardize formatting of headings and subheadings according to PLOS ONE style.	The formatting of all headings and subheadings has been standardized according to PLOS ONE style guidelines. This includes:<br>Numbered headings to reflect the hierarchy of sections and subsections.<br>Main section headings (e.g., Introduction, Materials and Methods, Results, Discussion) formatted in bold and title case with Arabic numerals (e.g., 1. Introduction). Second-level subheadings formatted in bold, sentence case, and numbered with decimals (e.g., 2.1 Study design). Third-level subheadings formatted in italics, sentence case, numbered with two decimals, and followed by a period, with the paragraph text continuing on the same line (e.g., 2.1.1 Inclusion criteria. Participants were selected based on...).<br>This structured, numbered format ensures clarity and consistency throughout the manuscript, in full compliance with PLOS ONE editorial standards.
Ensure all acronyms (e.g., JBI, OR) are defined on first use.	All acronyms used in the manuscript have been reviewed and are now defined at their first mention, in accordance with PLOS ONE guidelines. Examples include: JBI is defined as Joanna Briggs Institute at first use. OR is defined as odds ratio when it first appears. Any other acronyms (e.g., CI for confidence interval, SD for standard deviation) have also been expanded on initial mention. Subsequent uses of the acronyms appear without

	redefinition, maintaining clarity while ensuring readability for all audiences.
Please reword the conclusion to avoid repetition and to clearly articulate the key public health implications of your findings.	The conclusion has been reworded to eliminate repetition and to clearly emphasize the key public health implications of the findings. The revised version highlights the impact of diagnostic delays on patient outcomes and healthcare systems, and it underscores the need for improved awareness, early detection strategies, and strengthened clinical pathways. These changes provide a more concise and impactful summary that aligns with the broader public health significance of the study.
**Reviewer three comment**	**Author response**
ABSTRACT – "Anopheles" should be in italics.	Thank you, dear reviewer, we accept your comment and amendment has made accordingly.
INTRODUCTION<br>Line 74 - "Anopheles" must be in italics and started with a capital "A".<br>Lines 74, 75, 84 – "Anopheles" and "Plasmodium falciparum" must be in italics and capitalized.<br>Line 85 – "As evidenced by a national study,…" – Where is the study from?	Thank you, dear reviewer, we accept your comment and amendment has made accordingly. For line 85, it is from WHO 2019 malaria report.
Searching strategy – I considered it a good strategy and a good following entry terms<br>As this is not a longitudinal study, I suggest that the authors do not use the term "risk factors" anywhere in the text. Instead, use	Thank you, dear reviewer, we accept your comment and amendment has made accordingly.

"factors associated" with delayed treatment for malaria	
OUTCOME MEASUREMENTS - Line 146 – The explanation of OR is unnecessary	Thank you, so much dear reviewer. Amendment has made accordingly.
RESULTS<br>Line 192-193 – The authors wrote that "Of the total included studies, the majority, 28 (65.1%) were conducted in an urban setting". If 28 studies are 65.1%, the total number of studies should be 43. However, the number of studies included was 18. I may not have understood clearly, but I suggest checking this information.	Sorry dear reviewer, it was because of editorial error. Revision has made accordingly.
Don't the authors think that "child death" should be excluded from the factors associated with delayed treatment for malaria? This seems more related to a consequence rather than a cause.<br>Does the Education variable refer to the mother's education? I suggest making it clear.	Thank you for the valuable comments.<br>We agree that "child death" is more appropriately considered a consequence rather than a risk factor for delayed treatment. But in this context, it means that history of child death. Those mothers who had previous history of child death may have more likely to seek treatment early.<br>Regarding the education variable, yes, it refers specifically to the mother's level of education. We clarifed this in the text to avoid any ambiguity and ensure the reader clearly understands the variable's context. These adjustments will improve the accuracy and clarity of the findings.

Fig. 2 – I suggest deleting and keeping only Fig 4.<br>Fig 4 - I suggest putting the meaning of "ES" under the figures.<br>I didn't find Figure 6. Would Fig 7 be 6?	Dear reviewer your suggestion is fantastic. But fig 2 is summarize the objective one. So, delating this figure may omit the report of objective one. ES mean effect size. Figure 6 is available now.
All figures have two titles. I suggest choosing the title below the figures.<br>The number of figures (12!) is disproportionate to the magnitude of the results – it doesn't make sense. Authors can describe the results in the text and use a maximum of 2 to 3 figures to complement or clarify some relevant information. Another possibility would be a single figure referring to "Effect of the studied variables on malaria treatment delay" and presenting quadrants (a, b, c…) with the significant results including their respective significance levels.	We acknowledge that all figures currently include two titles, but the second title in the document is based on the recommendation or requirement of the journal.<br>The reason for 12 figures are because of reports are difficult to merged. Example, pooled prevalence and each factor never mixed up together. Funnel plot and forest plot never mixed up. The same is true for other figures and reports. The numbers of figures showed that how much the report is more comprehensive and exhaustive.
Line 350 – Check the word "drug" – Wouldn't it be "drug"?	Thank you, revision has made accordingly.
Reviewer four comment	Authors response
In L 187 and 191, authors need to justify the mention of "lowest prevalence of undiagnosed hypertension was reported in Sudan"? Are these sections from another paper?	Sorry dear reviewer. It is editorial error. Rather it means that delayed malaria treatment.
More than 90% of searched articles were removed due to duplicates. Please, authors need to explain in the methodology how they proceeded to remove duplicates. Additionally, authors should be consistent in writing: example 126,989 vs 119897	Thank you for your observation.<br>We acknowledge that more than 90% of the initially identified articles were excluded due to duplication. To address this, we revised the methodology section to clearly explain the process used for removing duplicates.

	Specifically, we used Covidence to automatically identify and remove duplicate records based on title, author, and publication year. A manual review was also conducted to ensure accuracy and remove any remaining duplicates not detected by the software.<br>How articles removed duplicates was also clearly explained in the PRISMA diagram.<br>The number of articles 126,989 is the total number of articles retrieved but 119,897 was the duplicated one.
The title of the study mentions "delay in seeking malaria diagnosis and treatment". However, the results showed only delayed treatment. Is the definition of delayed treatment in L 115-116 limited to clinical symptoms or after a test has confirmed Plasmodium infection?	Thank you for highlighting this important point.<br><br>You are correct that the title refers to delay in seeking both diagnosis and treatment, while the results primarily focus on delayed treatment. To address this inconsistency, we revised the title to accurately reflect the study's actual focus on treatment delay only,
In L 153, it would be better to rewrite the subheading as "Screening results" since you did not describe articles in this section.	We agree that the subheading in Line 153 would be more appropriately titled "Screening results with data extraction", as this section focuses on the identification and exclusion process rather than a description of the included articles. We revised the subheading accordingly to better reflect the content of the section and align with standard reporting practices in systematic reviews.
L 50: Keywords may be in alphabetical order	Thank you, dear reviewer, for your excellent view. Revision has made accordingly.
Write scientific names in italics in L 52-53.	Thank you, dear reviewer, for your excellent view. Revision has made accordingly.
In L 82, could the authors explain the importance of the clause "it took one month	Thank you for pointing this out.<br>We agree that the clause "it took one month to search articles" in Line 82 is redundant, as

to search articles" when the authors already mentioned the dates?	the specific date range for the search has already been provided. To maintain clarity and avoid unnecessary repetition, we will remove this clause from the manuscript (line 81 and 82).
L 213-214: The sentence "The pooled odds of delayed treatment for malaria among participants who had a history of drug side effects were 2.94 times more likely than the counterpart" is difficult to understand. More likely is not suitable for comparison of odds ratios between groups.	We agree that the sentence in Lines 213–214 is unclear and that the phrase "more likely" is not appropriate when interpreting odds ratios, as it can lead to confusion between odds and probabilities. We revised by higher odds.
For readability, authors may consider checking the punctuations throughout the manuscript.	Thank you, dear reviewer. We have made revision.
The authors should be consistent with tenses used in the manuscript. It would be better to use past tenses	Thank you, dear reviewer. We have made revision.
Some sentences and English used need to be improved in this manuscript, example L 274	Thank you, dear reviewer. We have made revision.
Check for typo errors and sentence clarity like in L 313-318 and other parts as well.	Thank you, dear reviewer. We have made revision accordingly.
L 319: "To do this, all the physicians, pharmacists, and caretakers should take responsibility for promoting the early treatment and diagnosis of malaria". Could the authors provide information on how this can be done?	Thank you, dear reviewer. To achieve this, physicians and pharmacists can engage in community education, provide clear counselling on the importance of early treatment, and ensure access to appropriate antimalarial drugs. Caretakers should be empowered through awareness campaigns to recognize early malaria symptoms and seek prompt care. Additionally, integrating malaria education into routine healthcare visits and collaborating with local health workers can support timely diagnosis and treatment

Thank you so much for your very impressive scientific contribution.
