# Supplementary material for: Prevalence and associated factors of delay in seeking malaria treatment among under five children in the Horn of Africa: A systematic review and meta-analysis
Source: PLoS One. 2025 Sep 26;20(9):e0333593. doi: 10.1371/journal.pone.0333593 (PMC12468861; doi:10.1371/journal.pone.0333593)
Supplement: Attachment — Submitted filename: Malaria treatment delay manscript_1.docx [file pone.0333593.s005.docx]

**Prevalence and determinants of delay in seeking malaria diagnosis and treatment among under five children in the Horn of Africa: A systematic review and meta-analysis**

Muluken Chanie Agimas^1^, Mekuriaw Nibret Aweke^2^, Berhanu Mengistu^2^, Lemlem Daniel Baffa^2^, Elsa Awoke Fentie^3^, Ever Siyoum Shewarega^3^, Aysheshim Kassahun Belew^2^, Esmael Ali Muhammad^2^

^1^Department of Epidemiology and Biostatistics, institute of public health, college of medicine and health science, university of Gondar, Gondar, Ethiopia.

^2^Department of human nutrition, institute of public health, college of medicine and health science, university of Gondar, Gondar, Ethiopia.

^3^Department of reproductive health, institute of public health, college of medicine and health science, university of Gondar, Gondar, Ethiopia.

**Authors address:**

Muluken Chanie Agimas (MCA): [mulukensrc12@gmail.com](mailto:mulukensrc12@gmail.com)

Mekuriaw Nibret Aweke (MNA): [mekunib@gmail.com](mailto:mekunib@gmail.com)

Berhanu Mengistu (BM): [berhanu5@yahoo.com](mailto:berhanu5@yahoo.com)

Lemlem Daniel Baffa (LDB): [lemmidan@gmail.com](mailto:lemmidan@gmail.com)

Elsa Awoke Fentie (EAF): [elsaawoke91@gmail.com](mailto:elsaawoke91@gmail.com)

Ever Siyoum Shewarega (ESS): [eversiyoum@yahoo.com](mailto:eversiyoum@yahoo.com)

Aysheshim Kassahun Belew (AKB): [aysheshim121@gmail.com](mailto:aysheshim121@gmail.com)

Esmael Ali Muhammad (EAM): [esmaelali34@gmail.com](mailto:esmaelali34@gmail.com)

Corresponding Author

MC: [mulukensrc12@gmail.com](mailto:mulukensrc12@gmail.com)

**Abstract**

**Introduction:** Malaria is a communicable parasitic disease caused by the female *Anopheles* mosquito. Malaria is a global public health problem, particularly in sub-Saharan African countries, where it accounts for 90% of global mortality and morbidity. To reduce the impact and complications associated with delayed treatment of malaria among children under five, comprehensive evidence about the magnitude and determinants of delayed treatment for malaria could be the solution. But there are no national-level studies in the Horn of Africa for decision-makers.

**Objective:** To assess the prevalence and determinants of delay in seeking malaria treatment and diagnosis among under-five children in the Horn of Africa.

**Method**: Published and unpublished papers were searched on Google, Google Scholar, PubMed/Medline, EMBASE, SCOPUS, and the published articles' reference list. The searching mechanism was established using Medical Subject Heading (MeSH) terms by combining the key terms of the title. To assure the quality of the included articles, Joana Brigg’s Institute critical appraisal checklist was used, and those articles with a total quality score greater than 50% were considered to have a low risk of bias. A sensitivity test was conducted to evaluate the heterogeneity of the studies. The visual funnel plot test and Egger’s and Begg’s statistics in the random effect model were done to evaluate the publication bias and small study effect. The I^2^ statistics were also used to quantify the amount of heterogeneity between the included studies.

**Results**: The pooled prevalence of delayed treatment for malaria among under-five children in the Horn of Africa was 48% (95% CI: 34%–63%). History of child death (OR =2.5, 95% CI: 1.73–3.59), distance >3000 meters (OR = 2.59, 95% CI: 2.03–3.3), drug side effect (OR = 2.94, 95% CI: 1.86-4.67), formal education (OR = 0.69, 95% CI: 0.49–0.96), middle income (OR = 0.42, 95% CI: 0.28–0.63), expensiveness (OR = 4.39, 95% CI: 2.49–7.76), and optimum cost (OR = 2.13, 95% CI: 1.41-3.2) for transport were factors associated with malaria treatment delay among children.

**Conclusion and recommendations:** In the Horn of Africa, for every two parents, approximately one parent delayed seeking treatment for malaria. Factors like fear of side effects from drugs, distance to reach the health facility >3000 meters, and expensive and optimum cost of transport were the risk factors for delayed treatment for malaria among children in the Horn of Africa. Whereas the middle level of income was the protective factor for delayed treatment for malaria among children, thus, expanding health education and improving financial and physical accessibility of health services are highly recommended to reduce treatment delays.

**Key word:** treatment delay, malaria, prevalence, determinants, children, horn of Africa

**Introduction**

Malaria is a communicable parasitic disease caused by female *Anopheles* mosquito [1, 2]. The plasmodium falciparum parasite is the most common responsible cause for malaria infection (3). Globally, children are the most vulnerable population and about 67% of all deaths of malaria are under five children [3]. Malaria is a global public health problem, particularly in sub-Saharan African countries, where it accounts for 90% of global mortality and morbidity [4]. Most malaria-related deaths can be reversed by prompt diagnosis and treatment . Even though there is a significant improvement towards the elimination of malaria in Africa, it is still the major public health concern in the tropical regions and in the poor health care systems of countries like the Horn of Africa [5].

The World Health Organization recommended that children confirmed with malaria get treatment within 24 hours and the timely access to anti-malaria medicine is very important to reverse the progression of plasmodium falciparum to severe form and death. In sub-Saharan African countries, the proportion of early treatment among children is very low [6].  As evidenced by a national study, between 2015 and 2018, about 36% of sub-Saharan African children under five did not receive malaria treatment [7]. In African countries, like Kenya, the meta-analysis report showed that only 5% of malaria patients are receiving treatment early (3). Although early treatment-seeking behavior is the most important strategy, long distance, lack of transport, and low income are the most common reasons for not receiving prompt treatment and diagnosis among children [8]. The other possible reason for delayed treatment for malaria among children is affordability, acceptability, and adequacy to meet expectations in quality of care [9, 10]. Unless children get malaria treatment early, it could be complicated into severe forms of malaria, disability, and death [11]. Today, several countries are trying to eliminate malaria by designing different initiatives like controlling the vectors, preventive treatments, and healthcare access, but in the sub-Saharan African countries, especially in the Horn of Africa, a significant proportion of children with malaria did not get any medical treatment. [12]. To reduce the impact and complications associated with delayed treatment of malaria among children under five, comprehensive evidence about the magnitude and determinants of delayed treatment for malaria could be the solution. But there are no national-level studies in the Horn of Africa for decision-makers. Therefore, the current study aimed to assess the prevalence and associated factors for delayed treatment of malaria among children in the Horn of Africa using a systematic review and meta-analysis.

**Methodology**

## Design

We have used and follow the Preferred Reporting Items for Systematic Reviews and Meta-Analysis Protocols (PRISMA-P 2015 Guidelines) to conduct this review [13].

## Searching strategy

Both published and unpublished articles were searched on search engines like Google, Google Scholar, PubMed/Medline, EMBASE, SCOPUS databases, and the published article’s reference list from January 17 to February 17, 2024. The medical subject heading (MeSH) terms with the key term of the title were used for searching. Missed articles from the search engines were checked for each retrieved article. The eight authors (Muluken Chanie Agimas, Aysheshim Kassahun Belew, Elsa Awoke Fentie, Lemlem Daniel Baffa, Esmael Ali Muhammad, Berhanu Mengistu, Ever Siyoum Shewarega, Mekuriaw Nibret Aweke) were involved in the independent searching of articles. To search for eligible articles from search engines, we used the following entry terms: "Delay and health care seeking OR treatment seeking OR risk factors OR determinants AND malaria AND under five children OR Ethiopia OR Djibouti OR Eritrea OR Somali OR Uganda OR Sudan OR South Sudan OR Kenya". All searched articles from different searching engines and methods were imported to EndNote X8 software for further article screening. To facilitate the screening process in the EndNote X8 software, we attach the PDF file to the EndNote X8 software. Before screening each article one by one using titles and abstracts, the first duplicate articles were removed. Paired authors screened the articles independently and cross-checked their reports on selected articles. The inconsistency among the authors was solved by discussion. Articles with full text were considered for systematic and meta-analysis. The quality of each selected article was assessed carefully by Joana Brigg’s Institute critical appraisal checklist [14] [15]. Articles with a total score of 50% and above were classified as having a low risk of bias; otherwise, they were classified as having a high risk of bias [15]. The quality of each paper was evaluated by each author, and discrepancies were solved through discussion.

**Eligibly criteria**

#### Inclusion criteria

### The eligibility criteria were formulated by the review questions. For the prevalence of delayed treatment for malaria, a CoCoPop (Condition, Context, and Population) review question was used. For risk factors of delayed treatment for malaria, the review question was PEO (Population, Ethics, and Outcome). Based on these review questions, those that fulfilled the following criteria were incorporated for analysis:

### Articles conducted in the horn of Africa on under five children and reported either prevalence of delayed treatment for malaria or associated factors of delayed treatment for malaria or both were included.

- .
- Articles conducted or published at any period and with English language were incorporated.

**Exclusion Criteria**

- Articles without full text after emailing the respective authors to obtain the full articles were excluded.
- Studies conducted with Cross sectional, case control, cohort and experimental study design were illegible for this review, case reports/case series studies, comments and opinions were excluded.

**Outcome measurement**

This systematic review and meta-analysis have two objectives. The first objective was to estimate the pooled prevalence of delayed treatment for malaria among children in the Horn of Africa. The second objective was to identify the risk factors for delayed treatment for malaria. Delayed treatment for malaria is defined as a treatment sought after the recommended 24-hour onset of malaria symptoms [4]. To measure the strength of the association between delayed treatment and associated factors, an odds ratio was used. The pooled odds ratio was calculated from the two-by-two table of the primary studies.

**Population**

Under five children in the Horn of Africa were the population for the current systematic review and meta-analysis.

**Data extraction**

## To extract the data from the selected articles, we have prepared an extraction check list using an Excel spreadsheet. Basically, the data extraction sheet was aimed at answering the question about the prevalence and associated factors of delayed treatment for malaria. The check list consists of the following data elements: publication year, authors, data collection tool, proportion of delayed treatment for malaria, standard error of the proportion, sample size, event (numbers of children with delayed treatment for malaria), study setting, study period, quality score, odds ratio for each predictor, log odds ratio for each predictor, upper confidence interval for odds ratio of each predictor, lower confidence interval for odds ratio of each predictor, ln of both upper confidence interval for odds ratio of each predictor, and standard error for odds ratio for each predictor. Additionally, we have used the JBI tool for data extraction. The data extraction was conducted by four pairs of authors, and the discrepancy between the authors was solved by group discussion. After double-checking the extracted data, the ready file was exported to STATA software version 17.

**Data analysis**

To analyze the pooled proportion and odds ratio by the extracted data, STATA software version-17 was used. To assess the index heterogeneity of the included studies the I^2^ statistics was calculated [16]. The I^2^ statistics value 25% was low heterogeneity whereas its value of 50%, and 75% was classified as medium and high heterogeneity between the studies respectively [17]. The statistical significance of the heterogeneity between the studies was evaluated by the p-value of I^2^ with the cut of point less than 0.05. To manage the heterogeneity between the studies, sensitivity analysis and subgroup analysis by study period and study setting was conducted. Additionally, the small study effect or the publication bias was critically assessed by graphical method the so called the visual funnel plot test. The second way to manage the small study effect bias was statistical method such as the Egger’s statistics. The random effect model was also used to estimate the pooled prevalence and odds ratio of delayed treatment for malaria with the level of confidence 95%. The pooled odds ratio with p-value of less than 0.05 was declared as a statistical predictor for delayed treatment for malaria among under five children in the horn of Africa. Findings such as the pooled prevalence delayed treatment for malaria and its determinants were reported by both the text and the forest plot.

**Results**

**Characteristics of the included articles**

Out of the total of 126,989 articles, 119897 were removed because of duplicates. About 810 records were screened, and 123 of them were assessed for eligibility. Finally, 18 potential studies have been included for systematic review and meta-analysis, as summarized in the PRISMA flow diagram **(Figure-1).**

**Characteristics of included studies**

All included studies were conducted using a cross-sectional study design. Of the total included studies, the majority, 28 (65.1%) were conducted in an urban setting. The total number of participants included or used for analysis was 64,156, and the minimum and maximum sample sizes used in the studies were 199 [18] and 18,430 [19] respectively. **(Table 1).**

**Table 1: characteristics of the included studies for delayed treatment of malaria in the horn of Africa.**

| S.No | Author | Study period | Country | Study setting | Quality | Sample size | Prevalence |
| --- | --- | --- | --- | --- | --- | --- | --- |
| 1 | Getahun et al [10]. | 2010 | Ethiopia | facility based | low risk of bias | 310 | 50% |
| 2 | Megersa Elias Gelchu [20] | 2021 | Ethiopia | facility based | low risk of bias | 565 | 84.8% |
| 3 | Daniel S et al [21] | 2018 | Ethiopia | facility based | low risk of bias | 326 | 40.5% |
| 4 | Ermias A et al [22] | 2014 | Ethiopia | facility based | low risk of bias | 302 | 50% |
| 5 | Olaka W et al [18] | 2019 | Kenya | facility based | low risk of bias | 199 | 59.8% |
| 6 | Mitiku and Assefa Malar J [23] | 2017 | Ethiopia | community based | low risk of bias | 491 | 61.3% |
| 7 | Bolanle. O et al [24] | 2022 | Ethiopia | community based | low risk of bias | 479 | 65.1% |
| 8 | Asfa Elnour et al [25] | 2017 | Sudan | community based | low risk of bias | 443 | 25.1% |
| 9 | Oryema-Lalobo, Michael [26] | 2009 | Uganda | community based | low risk of bias | 451 | 20.2% |
| 10 | Edgar M et al [19] | 2023 | Uganda | community based | low risk of bias | 18,430 | 63.6% |
| 11 | Kelly Marie Walters [27] | 2016 | Uganda | facility based | low risk of bias | 325 | 86.2% |
| 12 | Arthur M et al [28] | 2017 | Uganda | facility based | low risk of bias | 650 | Not reported |
| 13 | Wakgari D et al [29] | 2006 | Ethiopia | community based | low risk of bias | 2372 | 9.4% |
| 14 | Byakika-K et al [30] | 2009 | Uganda | facility based | low risk of bias | 200 | 34% |
| 15 | HALIMA M et al [31] | 1995 | Kenya | facility based | low risk of bias | 883 | 17% |
| 16 | Rosalind G.N. et al [32] | 1997 | Uganda | facility based | low risk of bias | 439 | 89.1% |
| 17 | Addisu T et al [33] | 2020 | Ethiopia | facility based | low risk of bias | 306 | 50% |
| 18 | David B.O &Christine A [34] | 2021 | Uganda | community based | low risk of bias | 236 | 17.8% |

**The pooled prevalence of delayed treatment for malaria in the horn of Africa.**

By using the random effect model, the pooled prevalence of delayed treatment for malaria among under five children in the Horn of Africa was 48% (95%CI: 34%–63%), and the heterogeneity among the studies was statistically significant (I^2^ = 98.82%, P-value < 0.001).  **(Figure 2).**

**Assessment of publication bias**

To evaluate the publication bias for the included articles, we used Egger’s statistical test and evidenced that there is no publication bias (β = -5.04, P-value = 0.74) **(Figure-3).**

**Figure-3: Funnel plot of delayed treatment for malaria among children in the horn of Africa**

**Handling heterogeneity**

In the random effect model of pooled prevalence, statistically significant heterogeneity was detected. To solve this problem, heterogeneity sensitivity analysis and subgroup analysis were performed.

**Sensitivity analysis**

To manage the influence of a single study in meta-analysis estimation (to assess the heterogeneity), the random effects model and sensitivity analysis were conducted, and there was no study that excessively influenced the overall pooled prevalence of undiagnosed hypertension (**Supporting file-1**).

**Subgroup analysis by country**

Again, to manage the heterogeneity, the subgroup analysis was conducted by country, and thus the highest prevalence of delayed treatment for malaria among children was observed in Uganda (52% (95%CI: 32%–72%) and the lowest prevalence of undiagnosed hypertension was reported in Sudan (13% (95%CI: 21%–29%) with an I^2^ =99.82% and a P-value <0.001) **(Figure-4).**

**Figure-4: The pooled prevalence of delayed treatment for malaria by country among children in the horn of Africa, 2023**

**Subgroup analysis by study setting**

Subgroup analysis was also carried out in the study setting as a source of heterogeneity. The highest prevalence of delayed treatment for malaria by study setting was observed in a facility-based study (56% (95% CI: 36%–76% with I2 =99.82%, P-value <0.001) **(Figure-5).**

**Figure-5: The pooled prevalence of delayed treatment for malaria by study setting among children in the horn of Africa, 2023**

**Subgroup analysis by study period**

Furthermore, subgroup analysis was carried out during the study period as a source of heterogeneity. Thus, the highest prevalence of delayed treatment for malaria by study period was among studies conducted after the COVID-19 pandemic: 58% (95% CI: 39%–76%, with I2 =99.82%, P-value <0.001) **(Figure-6).**

**Figure-6: The pooled prevalence of delayed treatment for malaria by study period among children in the horn of Africa, 2023**

**Factors associated with delayed treatment for malaria**

As briefly described in **Table 3**, distance, drug side effects, expensive and optimum transport costs, formal education, and middle income were significantly associated with delayed treatment for malaria among under-five children in the Horn of Africa. From the random effect model estimate, the odds of delayed treatment for malaria among participants who travelled >3000 meters to reach the health institution were 2.59 times higher than <3000 meters (OR = 2.59, 95% CI: 2.03-3.3, I^2^ =0%, P-value = 0.689) **(Table-3, Figure-7).** The pooled odds of delayed treatment for malaria among participants who had a history of drug side effects were 2.94 times more likely than the counterpart (OR = 2.94, 95% CI: 1.86-4.67, I^2^ =89.5%, P-value =0.002) **(Table-3, Figure-8).** The pooled odds of delayed treatment for malaria among participants who had formal education were reduced by 31% (OR = 0.69, 95% CI: 0.49–0.96, I2 = 91.4%, P-value<0.001) as compared to those who had no formal education **(Table-3, Figure-9)**. Regarding the cost of transport, participants who paid the expensive and optimum cost for transport were 4.39 (OR = 4.39, 95% CI: 2.49–7.76, I2 = 85.4%, P-value = 0.009) **(Table-3, Figure-10)** and 2.13 times (OR=2.13, 95% CI: 1.41-3.2 with I^2^=59.3%, P-value=0.117) **(Table-3, Figure-11)** more likely to delay the treatment of malaria, respectively. Additionally, the odds of malaria treatment delay among participants with a middle level of income were reduced by 58% (OR = 0.42, 95% CI: 0.28–0.63, I2 = 26.9%, P-value = 0.242) compared to those with a poor level of income **(Table-3, Figure-12)**.

**Figure-7: Effect of distance >3000 meter on malaria treatment delay among children in the horn of Africa.**

**Figure-8: Effect of drug side effect on malaria treatment delay among children in the horn of Africa.**

**Figure-9: Effect of formal education on malaria treatment delay among children in the horn of Africa.**

**Figure-10: Effect of expensive of transport cost on malaria treatment delay among children in the horn of Africa.**

**Figure-11: Effect of optimum transport cost on malaria treatment delay among children in the horn of Africa.**

**Figure-12: Effect of middle level income on malaria treatment delay among children in the horn of Africa.**

**Table 3. Summary of factors associated with delayed treatment for malaria among children in the horn of Africa.**

| Variable | | OR(95%CI) | Heterogeneity (I^2^,P-value) | Total studies | Sample size |
| --- | --- | --- | --- | --- | --- |
| Sex | Female | 2.69(0.98, 3.12) | 91.8, <0.001 | 2 | 865 |
|  | Male | 1 | 1 |  |  |
| Child death | Yes | 2.5 (0.73, 3.59) | 31.9, 0.23 | 3 | 918 |
|  | No | 1 | 1 |  |  |
| Distance | >3000 meter | 2.59 (2.03, 3.3) | 0%, 0.689 | 2 | 1286 |
|  | <3000 meter | 1 | 1 |  |  |
| Drug side effect | Yes | 2.94 (1.86, 4.67)* | 89.5%, 0.002 | 2 | 612 |
|  | No | 1 | 1 |  | 2846 |
| Cost of transport | Expensive | 4.39 (2.49, 7.76) | 85.4%, 0.009 | 2 | 636 |
|  | Optimum | 2.13 (1.41, 3.2) | 59.3%, 0.117 | 2 | 636 |
|  | Cheap | 1.9(0.98, 3.46) | 93.5, <0.001 | 2 | 636 |
|  | No cost | 1 | 1 |  |  |
| Education | Formal education | 0.69(0.49, 0.96)* | 91.4%, <0.001 | 3 | 3239 |
|  | No education | 1 | 1 |  |  |
| Polygamy | Yes | 3.03 (0.98, 4.05) | 0%, 0.359 | 2 | 960 |
|  | No | 1 | 1 |  |  |
| Unemployment | Yes | 1.23 (0.81, 1.89) | 96.2%, <0.001 | 2 | 871 |
|  | No | 1 | 1 |  |  |
| Income | Middle | 0.42(0.28, 0.63) | 26.9,0.242 | 2 | 2937 |
|  | Poor | 1 | 1 |  |  |

**Discussion**

Malaria treatment delay is a critical issue in Africa, especially in the Horn of Africa. It is the most common problem because of instability and poverty. To reverse this problem, studies to support decision-making are very important. But there are no national-level studies to estimate the pooled prevalence and factors that affect the malaria treatment delay of under-five children in the Horn of Africa. Therefore, in this study, an attempt has been made to assess the pooled prevalence of malaria treatment delay among under-five children in the Horn of Africa. Thus, the pooled prevalence of delayed treatment for malaria among under-five children in the Horn of Africa was 48% (95% CI: 34%–63%). It is obvious that, principally, all children with malaria should get treatment as soon as possible. But only 52% of under-five children in the Horn of Africa got prompt malaria treatment. This finding was lower than a study conducted in Myanmar, Southeast Asia, which was 64.7% (18). This can be justified by the variation in the study period. The other possible reason might be associated with the fact that the previous study was conducted exclusively among rural populations. The access to health services and health-seeking behavior among the rural population is relatively low. This leads to a delay in seeking malaria treatment [20]. But it was higher than a study conducted in South Eastern Nigeria 21.4% [35]. This might be because, in this study, we include those articles conducted after COVID-19 with the highest prevalence of delayed treatment for malaria. But a study conducted in south-eastern Nigeria was before the COVID-19 pandemic. This is because the COVID-19 pandemic had a negative effect on health service utilization. A subgroup analysis was carried out by the study period as a source of heterogeneity, and thus, the highest prevalence of delayed treatment for malaria by study period was among studies conducted after the COVID-19 pandemic (58%; 95% CI: 39%–76%). This might be because during the COVID-19 pandemic, the health institutions were focused on COVID-19 treatment and screening, but other health services were neglected.

Regarding factors associated with delayed treatment for malaria among under five children, as the distance to reach the health facility is far, the risk of delay in seeking diagnosis and malaria treatment is increased. The finding was supported by a study conducted in Yemen and India [36, 37] (17, 18). This might be associated with the fact that as the distance to reach the health facility increases, the tendency to access the health service becomes more challenged. Most of the time, the physical accessibility of the health service is a common problem for remote areas. This segment of the population is disadvantaged by poor media access, low health-seeking behavior, and the need to travel a long distance to access the health service. These all contribute to the delay in seeking treatment and diagnosis for malaria among under-five children in the Horn of Africa.

Another predictor that affects the treatment delay of malaria among under-five children in the Horn of Africa is educational level. Women with formal education have the lowest odds of delayed treatment for malaria compared to women who have no formal education. This finding was supported by a study conducted in Myanmar [38]. This might be associated with educational status, which strongly affects the health-seeking behavior of women. Women with inadequate literacy hinder the early seeking of health services because of a lack of information and knowledge about the signs and symptoms of malaria [39]. Education can also lead to more accurate health beliefs and knowledge, and thus to better lifestyle choices, better skills, and greater self-advocacy. Education improves skills such as literacy, develops effective habits, and may improve cognitive ability to decide the early treatment of malaria. Furthermore, educated women may be more able to understand their health needs, follow instructions, advocate for themselves and their families, and communicate effectively with health providers [40].

Regarding level of income, parents with a middle level of income have lower odds of delaying the treatment and diagnosis of malaria for their child than parents with a poor level of income. This finding was supported by a study conducted in China [41]. This might be associated with income having significant gradients in multiple measures of healthcare-seeking behavior. Wealth can give a person the freedom to choose how to live a healthy life without being constrained by financial problems to get health services early.

The cost of transport to reach the health institution is also another reason for delayed treatment for malaria among children in the Horn of Africa. This is because mothers of children with the highest and optimum cost of transportation to reach the health institutions have a higher risk of delayed treatment for malaria than women of children with no cost of transportation [10]. Furthermore, women who complained about the side effects of the malaria dug, which was ordered by the health professionals in the previous illness of the current child, were at higher risk of being delayed in seeking treatment for malaria among under five children in the Horn of Africa. This might be associated with inadequate health education and instructions about the general information and its side effects of malaria drugs by the health providers, which leads the women to believe that the side effects or adverse effects of malaria drugs are more harmful than their beneficial effects. Thus, adequate counselling and information for the women could improve the misunderstanding about the drug. To do this, all the physicians, pharmacists, and caretakers should take responsibility for promoting the early treatment and diagnosis of malaria. This study has an important implication for the body of knowledge by giving an insight into the factors contributing to delayed treatment for malaria among children. This knowledge is an important tool for decision-makers to design evidence-based strategies to reduce delay in malaria treatment and diagnosis in the Horn of Africa. This systematic review and meta-analysis had considerable strengths and limitations. Since it is the first-level study in the Horn of Africa, it provides important updated knowledge about why parents delay seeking treatment and diagnosis of malaria. Previous studies did not answer this question. Despite this strength, the current study includes only quantitative observational studies published in English, which may underestimate the pooled prevalence of delay in seeking treatment and diagnosis of malaria among under-five children in the Horn of Africa.

**Conclusion**

In the Horn of Africa, for every two parents, approximately one parent delayed seeking treatment for malaria. Factors like fear of side effects from drugs, distance to reach the health facility >3000 meters, and expensive and optimum cost of transport were the risk factors for delayed treatment for malaria among children in the Horn of Africa. Whereas the middle level of income was the protective factor for delayed treatment for malaria among children, thus, expanding health education and improving financial and physical accessibility of health services are highly recommended to reduce treatment delays.

**Ethical declaration**

**Ethical approval and consent to participate**

Because it was systematic review and meta-analysis, ethical consent was not applicable

**Consent for publication**

Not applicable.

**Data availability statement**

We, authors declare that all necessary data were available in the article.

**Conflict of interest**

No interest of conflict

**Source of funding**

There was no funding source for this research work.

**Acknowledgments**

The authors acknowledged the University of Gondar for facilitating trainings on meta-analysis.

**Author Contributions**

**Conceptualization**: Muluken Chanie Agimas, Lemlem Daniel Baffa, Aysheshim Kassahun Belew

**Data curation:** Muluken Chanie Agimas, Aysheshim kassahun Belew, Esmeal Ali Muhammad, Elsa Awoke Fentie

**Formal analysis**: Muluken Chanie Agimas, Aysheshim Kassahun Belew, Esmeal Ali Muhammad, Elsa Awoke Fentie

**Investigation**: Muluken Chanie Agimas, Aysheshim Kassahun Belew, Lemlem Daniel Baffa, Berhanu Mengistu, Esmael Ali Muhammad

**Methodology**: Berhanu Mengistu, Zufan Yiheyis Abriham,, Muluken Chanie Agimas, Elsa Awoke Fentie

**Software**: Muluken Chanie Agimas, Elsa Awoke Fentie, Esmael Ali Muhammad, Mekuriaw Nibret Aweke

**Supervision**: Muluken Chanie Agimas, Esmael Ali Muhammad

**Visualization**: Muluken Chanie Agimas, Lemlem Daniel Baffa, Esmael Ali Muhammad

**Writing – original draft**: Muluken Chanie Agimas, Berhanu Mengistu, Lemlem Daniel Baffa, Esmael Ali Muhammad, Elsa Awoke Fentie, Ever Siyoum Shewarega, Mekuriaw Nibret Aweke, Aysheshim Kassahun Belew

**Abbreviation and acronym**

CI…….Confidence Interval

COVID-19…..Corona Virus Disease 2019

JBI…… Joana Brigg’s Institute

OR……. Odds Ratio

PRISMA……. Preferred Reporting Items for Systematic Reviews and Meta-Analysis Protocols

**Reference**

1.	Bos, E.R., et al., *Disease and mortality in sub-Saharan Africa*. 2006: The World Bank.

2.	Berríos-Torres, S.I., et al., *Centers for disease control and prevention guideline for the prevention of surgical site infection, 2017.* 2017. **152**(8): p. 784-791.

3.	Giannone, B., et al., *Imported malaria in Switzerland,(1990–2019): A retrospective analysis.* 2022. **45**: p. 102251.

4.	White, N., S. Pukrittayakamee, and T. Hien, *WHO: Global technical strategy for malaria 2016–2030*. 2018.

5.	White, N., et al., *Erratum: Malaria (The Lancet (2014) 383 (723-735)).* 2014. **383**(9918).

6.	Stewart, B., C. Wild, and I.A.f.R.o.C.J.W.c. report, *Geneva: World Health Organization; 2014.* 2014.

7.	Organization, W.H., *World Malaria Report 2019: This Years Report at a Glance, WHO, Geneva, Switzerland, 2019*.

8.	Landier, J., et al., *The role of early detection and treatment in malaria elimination.* 2016. **15**: p. 1-8.

9.	Chuma, J., V. Okungu, and C.J.M.j. Molyneux, *Barriers to prompt and effective malaria treatment among the poorest population in Kenya.* 2010. **9**(1): p. 1-14.

10.	Getahun, A., K. Deribe, and A.J.M.J. Deribew, *Determinants of delay in malaria treatment-seeking behaviour for under-five children in south-west Ethiopia: a case control study.* 2010. **9**(1): p. 1-6.

11.	Castellani, J.S., *Delayed access to care for childhood malaria: Financial determinants and economic consequences.* 2020.

12.	Organization, W.H., *Update on the E-2020 initiative of 21 malaria-eliminating countries: report and country briefs*. 2018, World Health Organization.

13.	Moher, D., et al., *Preferred reporting items for systematic review and meta-analysis protocols (PRISMA-P) 2015 statement.* Systematic reviews, 2015. **4**(1): p. 1-9.

14.	Munn, Z., et al., *Methodological guidance for systematic reviews of observational epidemiological studies reporting prevalence and cumulative incidence data.* International journal of evidence-based healthcare, 2015. **13**(3): p. 147-153.

15.	Fan, S.-J., et al., *Ambient air pollution and depression: a systematic review with meta-analysis up to 2019.* Science of The Total Environment, 2020. **701**: p. 134721.

16.	Higgins, J.P. and S.G. Thompson, *Quantifying heterogeneity in a meta‐analysis.* Statistics in medicine, 2002. **21**(11): p. 1539-1558.

17.	J, I., *Interpretation of tests of heterogeneity and bias in metaanalysis. .* J Eval Clin Prac, 2008. **14(5)**: p. 951 –7.

18.	Olaka, W., et al., *Home based care practices contribute to delayed health care seeking for children under five years with fever in malaria endemic areas of Western Kenya.* 2019.

19.	Mulogo, E., et al., *Promptness in seeking treatment from Village Health workers for children under five years with malaria, diarrhoea and pneumonia in rural southwestern Uganda.* 2023. **22**(1): p. 198.

20.	Elias, M., *Early Treatment-Seeking Behaviour for Malaria and Its Associated Factors Among Caregivers of Under-Five Children With Fever in Abaya District, West Guji Zone, Oromia, Ethiopia*. 2021, HUCMHS.

21.	Shiferaw, D., M. Geremew, and A.J.M. Kumalo, *Determinants of Delay in Malaria Treatment Seeking for Under-Five Children with Malaria Attending Health Centers of Bench-Maji Zone, South-Western Ethiopia Case-Control Study.* 1930. **1**: p. 2016.

22.	Turuse, E.A., K.A. Gelaye, and T.K.J.H. Beyen, *Determinants of delay in malaria prompt diagnosis and timely treatment among under-five children in Shashogo Woreda, Hadiya Zone, Southern Ethiopia: a case control study.* 2014. **2014**.

23.	Mitiku, I. and A.J.M.j. Assefa, *Caregivers’ perception of malaria and treatment-seeking behaviour for under five children in Mandura District, West Ethiopia: a cross-sectional study.* 2017. **16**(1): p. 1-10.

24.	Olapeju, B., et al., *Psychosocial factors associated with malaria care-seeking in rural Ethiopia.* 2022. **22**(1): p. 1460.

25.	Elfaki, A.E.M., M.K.M. Elnimeiri, and W.E.M.J.I. Elfakey, *Management seeking behavior of malaria among mothers of under-five year’s children in Damazin Locality, Blue Nile State, Sudan.* 2017. **6**: p. 14-19.

26.	Oryema-Lalobo, M.J.E.A.J.o.P.H., *Community health seeking practices for the management of malaria of the under-five in Bugiri District, Uganda.* 2009. **6**(2).

27.	Walters, K.M., *Treatment-seeking behavior and delay in children with severe malaria in Uganda: results of a Markov analysis*. 2016.

28.	Mpimbaza, A., et al., *Demographic, socioeconomic, and geographic factors leading to severe malaria and delayed care seeking in Ugandan children: a case–control study.* 2017. **97**(5): p. 1513.

29.	Deressa, W., et al., *Household and socioeconomic factors associated with childhood febrile illnesses and treatment seeking behaviour in an area of epidemic malaria in rural Ethiopia.* 2007. **101**(9): p. 939-947.

30.	Byakika-Kibwika, P., G. Ndeezi, and M.J.A.h.s. Kamya, *Health care related factors associated with severe malaria in children in Kampala, Uganda.* 2009. **9**(3): p. 206.

31.	Mwenesi, H., et al., *Child malaria treatment practices among mothers in Kenya.* 1995. **40**(9): p. 1271-1277.

32.	Kassam, R., et al., *Patterns of treatment-seeking behaviors among caregivers of febrile young children: a Ugandan multiple case study.* 2016. **16**: p. 1-24.

33.	Shumerga, A.T., et al., *Determinants of delay in seeking malaria treatment for under-five children at Gambella Town, Southwest Ethiopia: a case-control study.* 2020. **2020**.

34.	Odimbe, D.B. and C. Atuhairwe, *Factors Influencing Caregivers’ Health Seeking Behavior for Malaria Treatment of Children Under 5 Years in Busia Municipality, Uganda.* 2021.

35.	Chukwuocha, U., et al., *Social determinants of malaria treatment seeking time by mothers for children (0–5 years) in South Eastern Nigeria.* 2015. **2**(154): p. 2.

36.	Al‐Taiar, A., et al., *Who develops severe malaria? Impact of access to healthcare, socio‐economic and environmental factors on children in Yemen: a case‐control study.* 2008. **13**(6): p. 762-770.

37.	Das, A. and T.S.J.M.j. Ravindran, *Factors affecting treatment-seeking for febrile illness in a malaria endemic block in Boudh district, Orissa, India: policy implications for malaria control.* 2010. **9**: p. 1-10.

38.	Aung, T., et al., *Improving the quality of paediatric malaria diagnosis and treatment by rural providers in Myanmar: an evaluation of a training and support intervention.* 2015. **14**: p. 1-9.

39.	Paasche-Orlow, M.K. and M.S.J.A.j.o.h.b. Wolf, *The causal pathways linking health literacy to health outcomes.* 2007. **31**(1): p. S19-S26.

40.	Goldman, D.P. and J.P.J.P.o.t.N.A.o.S. Smith, *Can patient self-management help explain the SES health gradient?* 2002. **99**(16): p. 10929-10934.

41.	Li, X., et al., *Effect of socioeconomic status on the healthcare-seeking behavior of migrant workers in China.* PLoS One, 2020. **15**(8): p. e0237867.
